# The Determination of Trace Elements in Uranium Oxide (U_3_O_8_) by Inductively Coupled Plasma Emission Spectrometry and Graphite Furnace Atomic Absorption Spectrometry

**DOI:** 10.6028/jres.093.116

**Published:** 1988-06-01

**Authors:** Patricia M. Santoliquido

**Affiliations:** U.S. Department of Energy, New Brunswick Laboratory, 9800 South Cass Avenue, Argonne, IL 60439

The observations and data presented here are drawn from a larger project which has as its objective the certification of a set of reference materials, known as CRM 123 (1-7), for the 18 trace elements contained in each of the seven levels which make up the set. Level 7 is high purity U_3_O_8_. Portions from the bulk of this same material were used as the base material for all the other concentration levels, which were made by adding known concentrations of impurity elements. CRM 123 (1-7) is a replacement for a previous reference material of the same type called CRM 98 (1-7). When that reference material was certified the workhorse methods were flame atomic absorption and spectrophotometric methods. Both graphite furnace atomic absorption (GFAA) and inductively coupled plasma emission spectrometry (ICP) had become established methods of analysis since then. The characterization of CRM 123 (1-7) provided an opportunity to take advantage of the greater sensitivity these newer methods could provide. In general the two techniques are complementary rather than competitive. A look at the characteristics of each method shows why we should have different expectations for each.

GFFA has a small linear dynamic range. The sensitivity for uranium is low; therefore, direct analysis without prior separation of uranium is possible. Volatile elements are the most sensitive analytes, so this should be a good method for cadmium, for example. A search of the literature [1–4] showed that the trail had already been blazed and that accurate results could be achieved in a 0.1 *N* nitric acid medium as long as the standards are matrix-matched for uranium as well. The amount of uranium present affects sensitivity. For cadmium there was no particular pattern for the sensitivity changes. For manganese, chromium, and aluminum there was a gradual decrease in sensitivity with more than 100 micrograms of uranium present (fig. 1). The change for magnesium and copper was much more dramatic (fig. 2). Copper and magnesium were determined using pyrocoated tubes; the other elements were determined using regular graphite tubes.

In contrast to GFAA, ICF has a large linear dynamic range and is very sensitive for uranium and the more refractory elements that GFAA does poorly. The large linear dynamic range means that the same sample size may be used for many different concentration levels. The high sensitivity for uranium is a major problem and 1,458 ICP spectral lines have been identified for uranium [5]. Not only are direct spectral interferences a problem but with uranium as the matrix the wings from these lines are so high that they completely obscure any trace elements present. Separation of the uranium prior to measurement of the trace elements is essential. The literature [6–9] shows that a number of different approaches have been successful. A tributyl phosphate extraction from a 1.6 *N* nitric acid medium was chosen for this work. This proved to be successful for many elements. In separate analyses a cupferron separation was used for zirconium and an α-benzoin oxime extraction was used for molybdenum.

Both GFFA and ICP are relatively straightforward techniques. For GFAA the problem areas are environmental magnesium and chromium memory. For ICP it is spectral interference and structured background that we must watch out for. Background structure becomes increasingly important at lower concentrations—not only for wavelength selection but also for choosing background correction positions. By GFAA there are six elements that can be determined directly in uranyl nitrate solution: cadmium, chromium, aluminum, copper, magnesium, and manganese. By ICP after appropriate separations a larger group may be determined. These elements are aluminum, calcium, iron, nickel, copper, molybdenum, sodium, manganese, vanadium, zirconium, and magnesium. The method using GFFA is better for cadmium and chromium while the method using ICP is better for calcium, iron, nickel, molybdenum, sodium, vanadium, and zirconium. There are four elements, however, that are done equally well by each 5,2 method. These elements are aluminum, magnesium, copper, and manganese. Results for these elements are given in tables 1 to 4. Neither technique showed a systematic bias throughout all concentration levels, although for some levels of copper and manganese GFAA exhibited a small positive bias.

## Figures and Tables

**Figure 1:**
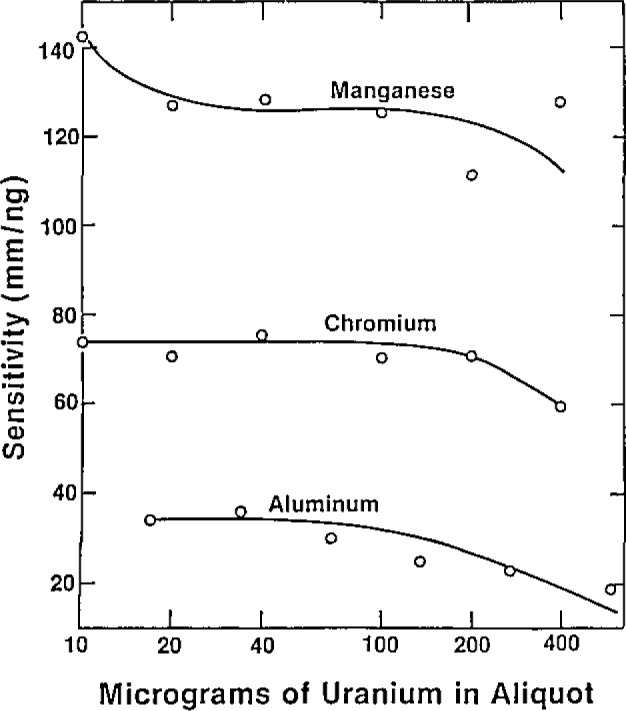
Influence of uranium concentration on sensitivity For aluminum, chromium and manganese by GFAA.

**Figure 2:**
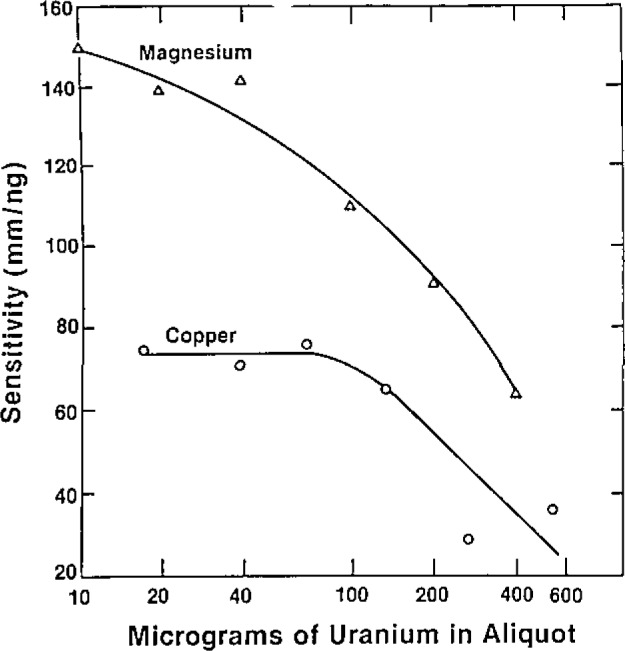
Influence of uranium concentration on sensitivity for copper and magnesium by GFAA.

**Table 1:** Aluminum determinations, μg/g U, mean ±s_x_, n= 10

Level	Added to base	ICP	GFAA
123-1	200	207.5±2.3	202.6±7.3
123-2	100	97.6±1.1	99.1±3.3
123-3	50	47.6±0.6	51.2±1.9
123-4	20	19.9±0.4	23.3±1.2
123-5	10	10.1±0.2	12.1±1.0
123-6	5	5.0±0.1	6.1±0.4
123-7	0	<0.9	<2.2

**Table 2:** Magnesium determinations, *μ*g/g U, mean ±s_x_, n=10

Level	Added to base	ICP	GFAA
123-1	100	100.8±1.3	103.9±5.8
123-2	50	50.3±1.7	51.2±0.2
123-3	20	20.3±0.2	20.3±0.8
123-4	10	10.7±0.7	11.4±0.6
123-5	5	5.6±0.1	5.5±0.4
123-6	2	3.4±0.3	2.5±0.2
123-7	0	1.8±0.4	<1.0

**Table 3:** Copper determinations, *μ*g/g U, mean ± s_x_, n=10

Level	Added to base	ICP	GFAA
123-1	50	49.6± 1.3	56.0±2.1
123-2	25	23.4±0.4	27.7±0.5
123-3	10	8.8±0.2	12.9±0.5
123-4	5	5.0±0.1	6.8±0.6
123-5	2.5	2.70±0.05	2.5±0.1
123-6	1	1.18±0.06	1.2±0.1
123-7	0	0.20±0.07	<0.6

**Table 4:** Manganese determinations, *μ*g/g U, mean ± s_x_, n=10

Level	Added to base	ICP	GFAA
123-1	50	51.1±1.5	52.7±1.3
123-2	25	25.4±0.4	29.4±0.6
123-3	10	10.9±0.2	12.7±0.3
123-4	5.0	5.45±0.07	5.7±0.2
123-5	2.5	2.90±0.04	3.25±0.08
123-6	1.0	1.14±0.03	1.33±0.08
123-7	0	0.30±0.02	0.24±0.03
